# Associations between wives' and husbands' attitudes towards women's economic participation and depressive symptoms, poor subjective health, and unemployment status in married women: A Korean longitudinal study (2014–2020)

**DOI:** 10.1016/j.ssmph.2022.101275

**Published:** 2022-10-22

**Authors:** Seong-Uk Baek, Jin-Ha Yoon, Jong-Uk Won

**Affiliations:** a Department of Occupational and Environmental Medicine, Severance Hospital, Yonsei University College of Medicine, Seoul, South Korea; b The Institute for Occupational Health, Yonsei University College of Medicine, Seoul, South Korea; c Graduate School, Yonsei University College of Medicine, Seoul, South Korea; d Graduate School of Public Health, Yonsei University College of Medicine, Seoul, South Korea; e Department of Preventive Medicine, Yonsei University College of Medicine, Seoul, South Korea

**Keywords:** Gender role attitude, Depressive symptom, Subjective health, Unemployment, Economic participation

## Abstract

**Introduction:**

Previous research has investigated the relationship between an individuals' gender role attitudes (GRAs) and their psychological health. We hypothesized that holding traditional GRAs or having a husband who holds traditional GRAs may adversely affect a woman's health.

**Methods:**

Data were obtained from a nationally representative longitudinal survey. Women's negative attitudes towards women's economic participation and husbands' negative attitudes towards their wives' economic participation were measured. The associations between the two and depressive symptoms, poor subjective health, and unemployment status in married women was estimated using a generalised estimating equation. Odds ratios (OR) and 95% confidence intervals (CIs) were calculated.

**Results:**

Women's negative attitudes towards women's economic participation was associated with depressive symptoms (OR [95% CI]: 1.19 [1.09–1.31]), poor subjective health (1.14 [1.04–1.25]) and unemployment status (1.10 [1.05–1.15]) in married women. In addition, there were significant associations between husbands' negative attitudes towards their wives working and depressive symptoms (1.41 [1.23–1.60]), poor subjective health (1.69 [1.48–1.92]), and unemployment (1.80 [1.69–1.92]) in their wives. The effect was strongest when both wives and their husbands have negative attitudes. In addition, the models considering cumulative years of negative attitudes showed that wives holding negative attitudes towards women's economic participation for 3 years or more was associated with depressive symptoms (1.70 [1.42–2.04]), poor subjective health (1.28 [1.04–1.57]), and unemployment status (1.39 [1.22–1.58]). Similarly, husbands' holding 3 years or more of negative attitudes towards their wives' economic participation was associated with depressive symptoms (1.32 [1.02–1.72])), poor subjective health (1.81 [1.40–2.35]), and unemployment status (9.02 [7.97–10.21]) in their wives.

**Conclusions:**

Our results show that one's own or one's husband's attitude towards women's economic participation affects not only the employment status of married women but also their mental and subjective health. Policymakers should implement policies that encourage positive attitudes towards women's economic activities.

## Highlights

• Traditional gender role attitudes increase the odds of depressive symptoms.
• Traditional gender role attitudes increase the odds of poor subjective health.
• Husbands who oppose their wives working increase their wives' risk for depression.
• Husbands who oppose their wives working increase their wives' risk for poor health.
• A longer duration of traditional or negative attitudes increases the impact.

## 1 Introduction

Gender roles are defined as social expectations regarding the behaviours and perceptions of each sex (Eagly & Wood, 1991). According to social role theory, socialization facilitates acceptance of traditional gender roles by encouraging men and women to behave in ways expected of their sex (Eagly, 1997). Individuals who hold traditional gender role attitudes (GRAs) believe that men should serve as the ‘breadwinners’ of the family and that women should serve as the caretakers of the family and children. Thus, traditional gender roles constrain women from working outside the home (Vella, 1994, pp. 191–211). This situation has been highlighted as a major cause of gender disparities in labour force participation (Kumari, 2018). In the context of Asia, where conservative attitudes towards women working are prevalent (Chia et al., 1994), the gender gap is even greater. For example, the average female labour participation rate of the Organisation for Economic Co-operation and Development countries is 63.8%, whereas for Korea, the rate is only 55.6% (Statistics Korea, 2022).

Numerous studies have investigated the relationship between GRAs and various individual behaviours and psychological health. It has been found that conformity to masculine or feminine norms affect individual health behaviours. For example, men who conform to masculine norms have a tendency to consume more meat and women who conform to feminine norms have a higher risk of eating disorders due to their striving for thinness (Fleming & Agnew-Brune, 2015; Sloan et al., 2015). In addition, traditional gender identity has been found to be associated with an increased risk of depression in women (Rovira et al., 2022). Furthermore, studies have shown that gender role stress occurs when an individual's preferences or behaviours conflict with one's own gender-related stereotypes, and this stress is associated with psychiatric problems such as eating disorders and depression (Bekker & Boselie, 2002; Kuehner, 2003).

Marital relationships are a major determinant of the physical and mental health of married women (Choi & Marks, 2008; Robles, 2014). Although it has been well documented that holding traditional GRAs is associated with psychological distress in women (Brody et al., 2014; Hunt et al., 2006; Jaehn et al., 2020), the crossover effect of husbands' GRAs on their wives' health is largely unexamined. Nonetheless, some studies have found that a spouse's traditional GRAs can worsen the marital satisfaction and psychological well-being of women (Amato & Booth, 1995; Seol et al., 2022).

From this perspective, holding traditional GRAs or having a husband who holds traditional GRAs may adversely affect a woman's health. Married women's gender role stress may be intensified by their spouses, especially when their husbands have negative attitudes towards them working. In contrast, if the husband has a supportive attitude towards his wife working, gender role conflicts may be reduced. Hu et al. revealed that when wives and husbands were not aligned in terms of GRAs, the role overload of wives could be induced which can adversely affect women's mental health (Hu et al., 2021; Shimazu et al., 2013). A previous Korean study reported that the employment status of married women was strongly affected by their husbands' attitudes towards their economic participation (Gwak & Choi, 2015). However, to the best of our knowledge, there is no research on the impact of husbands' attitudes towards their wives' economic participation on married women's health.

Thus, our study aimed to explore the following hypotheses: (i) Holding negative attitudes towards women's economic participation is associated with depressive symptoms, poor subjective health, and unemployment status among married women. (ii) Husbands' negative attitudes towards their wives' economic participation are associated with depressive symptoms, poor subjective health, and unemployment status in married women.

## 2 Materiel and methods

### 2.1 Study samples

This study used raw data from the Korean Longitudinal Survey of Women and Families (KLoWF). The KLoWF is an ongoing nationally representative panel study conducted since 2007, and the data are collected every other year. The KLoWF sampled study participants using two-stage stratified sampling based on the National Population and Housing Census conducted in 2005. Thus, the participants in the KLOWF surveys were representative of the population of Korean women. Professional interviewers conducted the survey via computer-assisted personal interviewing or tablet-assisted personal interviewing. Participants from 2014 to 2020 (waves 5–8) were included in the current study because the current form of the survey items on depressive symptoms was introduced in 2014.

Of the initial 7745 participants in 2014, we included only married women, leaving 5755 participants. Next, to establish balanced panel data, we limited the sample to those who participated in all four surveys (waves 5–8) as married women, leaving 4420 participants with 17,680 observations. After excluding six participants with missing values, our final study sample included 4414 women with a total of 17,656 observations (Table S1). Therefore, our study is a complete case analysis. The baseline characteristics of the included and excluded participants were shown in Table S2. The flowchart of the selection of study participants was shown in Fig. S1. Sensitivity analyses were also conducted based on the unbalanced panel data which included 20,480 observations of 5748 participants with no missing values, who answered one or more survey. The same association was observed in sensitivity analyses (results not shown).

### 2.2 Data availability and ethics statement

The KLoWF data are available, and researchers can download the raw data upon request (https://klowf.kwdi.re.kr). The raw KLoWF data do not include any personal information. This study was reviewed and approved by the Institutional Review Board of Yonsei Health System (approval number: 4-2022-0691).

### 2.3 Main variables

#### 2.3.1 Main independent variables

One's own attitudes towards women's economic participation was assessed with the following question: ‘What do you think about the following sentence? - It is ideal for men to work and women to take care of their families?’ The response was measured on a 4-point Likert scale (‘strongly agree’ [1], ‘somewhat agree’ [2], ‘somewhat disagree’ [3], ‘strongly disagree’ [4]). We classified those who responded either ‘strongly agree’ or ‘somewhat agree’ as having negative attitudes towards women's economic participation.

Next, the husband's attitudes towards the wife's economic participation (respondent) were assessed with the following question: ‘What does your husband think about your participation in economic activities?’ The response was measured on a 5-point Likert scale (‘strongly oppose’ [1], ‘slightly oppose’ [2], ‘neither oppose nor approve’ [3], ‘slightly approve’ [4], ‘strongly approve’ [5]). We classified those who responded either ‘strongly oppose’ or ‘slightly oppose’ as having husbands with negative attitudes towards their wives' economic participation.

#### 2.3.2 Dependent variables

We used the Korean version of the 10-item Centre for Epidemiological Studies Depression Scale (CES-D-10) to measure depressive symptoms. The reliability and validity of the Korean version of the questionnaire have also been investigated in a previous study and are widely used as efficient instruments to measure subjects' depressive symptoms (Shin, 2011). For each item on the questionnaire, the responses are based on a 4-point Likert scale, and the total score ranges from 0 to 30. Cronbach's alpha was 0.86 in the current study. A cut-off value of 10 has been validated in multiple studies (Irwin et al., 1999; Shin, 2011). Thus, a CES-D-10 score of 10 or higher was considered to indicate clinically significant depressive symptoms.

Subjective health was measured using the following question: ‘How do you feel about your current health?’ The response was measured on a 5-point Likert scale (‘very bad’ [1], ‘fairly bad’ [2], ‘so-so’ [3], ‘fairly good’ [4], and ‘very good’ [5]). We classified those who responded either ‘very bad’ or ‘fairly bad’ as having poor subjective health.

Employment status was classified as employed worker, self-employed, unpaid family worker, or unemployed. The term ‘unemployed’ refers to those who did not fall into any of the above categories and have any jobs and is used as one of the dependent variables.

### 2.4 Covariates

Age group, education, household income, number of children, marital satisfaction, health-related behaviours (regular physical activity, smoking status, problematic alcohol use), and survey year were considered as confounders in our analyses. In the analyses of the relationship between negative attitudes and health outcomes (depressive symptoms and poor subjective health), the employment status of the participants was additionally included as a covariate. The age groups were categorised as <40, 40–49, 50–59, and ≥60 years. Education level was categorised as having completed high school or lower, college, and none. The income categories were classified into quartiles based on household income. The number of children was categorised as 0, 1, and 2 or more children under the age of 20 years. Marital satisfaction was assessed using the following question: ‘How do you feel about your current marital life with your husband’? The respondents answered on a scale of 1 (‘very unhappy’) to 10 (‘very happy’), and the score was used as a continuous variable. Physical activity was assessed using the following question: ‘Have you recently performed vigorous physical activity for 10 min or more that made you feel tired or made you breathe more heavily? If yes, please answer how many days you did during the past week’. We defined those who engaged in physical activity 1 day or more as those who had performed a ‘physical activity’. Regarding smoking status, study subjects were classified as current or past smokers and never smokers. Problematic drinking use was defined as a CAGE score of 1 or higher (Ewing, 1984). We adjusted the survey year by including binary dummy variables for each year.

### 2.5 Statistical analysis

For the descriptive analysis, the chi-square test and *t*-test were performed to compare the characteristics of the study samples by one's own or husband's attitude towards women's/wives' economic participation. For the regression analysis, the generalised estimating equation (GEE) with autoregressive working correlation was employed to estimate odds ratios (ORs) and 95% confidence intervals (CIs). To select the working correlation structure for GEEs, the correlation between repeatedly measured binary outcome within individuals was analyzed using a loreogram (Heagerty & Zeger, 1998; Iannarilli et al., 2019). The primary analysis was analyzed using the autoregressive structure, and we checked the robustness of our finding using exchangeable and unstructured correlation structure in the sensitivity analysis (Tables S3–S6). First, we estimated the odds ratio for each variable. Next, in order to estimate the combined effect of the two main independent variables, these two variables were grouped and reclassified as ‘one's own negative attitudes (−) and husband's negative attitudes (−)’, ‘one's own negative attitudes (+) and husband's negative attitudes (−)’, ‘one's own negative attitudes (−) and husband's negative attitudes (+)’, and ‘one's own negative attitudes (+) and husband's negative attitudes (+)’. The number of years of experiencing each negative attitude was also measured to explore the cumulative effect. Within the observation period, the cumulative years (waves) of experiencing each negative attitude until each year of concern were classified as (i) 0, (ii) 1, (iii) 2, and (iv) ≥ 3 years. Finally, to estimate the effect of the sequential experience of each GRA, negative attitudes of the previous year (*t-1*) and the concerned year (*t*) were grouped and reclassified as follow: (i) ‘No → No’, (ii) ‘Yes → No’, (iii) ‘No → Yes’, and (iv) ‘Yes → Yes’. R (version 4.2.0; R Foundation for Statistical Computing, Vienna, Austria) was used for all statistical analyses and visualisation.

## 3 Results

Table 1 presents the baseline sociodemographic characteristics of pooled observations by one's own and husband's attitudes towards women's/wives' economic participation. Women who held negative attitude women's economic participation had a higher proportion of those aged 50 or older and low-income groups (Q1 or Q2) than those who did not. Among those who held negative attitudes towards women's economic participation, there was a lower proportion of subjects who had completed college compared with those who held positive attitudes (15.9% vs. 20.3%, P < 0.001). In terms of husbands' attitudes, the majority of those whose husbands had negative attitudes towards their wives' economic participation were older women. However, no significant difference was observed in education level. In addition, those who had negative attitudes showed no significant difference in terms of marital satisfaction compared with those with positive attitudes (6.8 vs. 6.8, P = 0.345). Those whose husbands opposed their participation in economic activities showed greater marital satisfaction than those whose husbands did not (6.9 vs. 6.7, P < 0.001). Table S1 presents the sociodemographic characteristics of the study sample for each survey year.

**Table 1:** Baseline sociodemographic feature among pooled observations by one's own and husband's negative attitude toward women/wife's economic participation (Number of total observations = 17,656).

	One's own negative attitude			Husband's negative attitude		
	Yes (N = 8431)	No (N = 9125)	P value	Yes (N = 2808)	No (N = 14,848)	P value
Age group						
< 40	796 (9.4%)	970 (10.5%)	<0.001	354 (12.6%)	1412 (9.5%)	<0.001
40-49	2399 (28.5%)	3293 (35.7%)		765 (27.2%)	4927 (33.2%)	
50-59	2370 (28.1%)	2526 (27.4%)		647 (23.0%)	4249 (28.6%)	
≥ 60	2866 (34.0%)	2436 (26.4%)		1042 (37.1%)	4260 (28.7%)	
Income (in quartile)						
Q1	2432 (28.8%)	2073 (22.5%)	<0.001	906 (32.3%)	3599 (24.2%)	<0.001
Q2	2294 (27.2%)	2243 (24.3%)		733 (26.1%)	3804 (25.6%)	
Q3	1934 (22.9%)	2287 (24.8%)		594 (21.2%)	3627 (24.4%)	
Q4	1771 (21.0%)	2622 (28.4%)		575 (20.5%)	3818 (25.7%)	
Unemployment						
Employed worker	2059 (24.4%)	3120 (33.8%)	<0.001	248 (8.8%)	4931 (33.2%)	<0.001
Self-employed	924 (11.0%)	1396 (15.1%)		109 (3.9%)	2211 (14.9%)	
Unpaid family worker	1506 (17.9%)	1357 (14.7%)		100 (3.6%)	2763 (18.6%)	
Unemployed	3942 (46.8%)	3352 (36.3%)		2351 (83.7%)	4943 (33.3%)	
Education						
College	1343 (15.9%)	1874 (20.3%)	<0.001	536 (19.1%)	2681 (18.1%)	0.161
High school or under	6861 (81.4%)	7196 (78.0%)		2202 (78.4%)	11,855 (79.8%)	
None	227 (2.7%)	155 (1.7%)		70 (2.5%)	312 (2.1%)	
Number of children						
No	5185 (61.5%)	5150 (55.8%)	<0.001	1687 (60.1%)	8648 (58.2%)	0.005
One	1189 (14.1%)	1471 (15.9%)		367 (13.1%)	2293 (15.4%)	
Two or more	2057 (24.4%)	2604 (28.2%)		754 (26.9%)	3907 (26.3%)	
Relationship with husband (range: 1–10)						
Mean (SD)	6.8 (1.5)	6.8 (1.6)	0.345	6.9 (1.5)	6.7 (1.6)	<0.001
Regular physical activity						
No	6200 (73.5%)	6836 (74.1%)	0.403	1939 (69.1%)	11,097 (74.7%)	<0.001
Yes	2231 (26.5%)	2389 (25.9%)		869 (30.9%)	3751 (25.3%)	
Smoking						
Never	8365 (99.2%)	9124 (98.9%)	0.039	2780 (99.0%)	14,709 (99.1%)	0.842
Current/past	66 (0.8%)	101 (1.1%)		28 (1.0%)	139 (0.9%)	
Problematic alcohol use						
No	8334 (98.8%)	9103 (98.7%)	0.335	2791 (99.4%)	11,097 (74.7%)	0.001
Yes	97 (1.2%)	122 (1.3%)		17 (0.6%)	202 (1.4%)	

*Chi-square test or Student's t-test.

Table 2 presents the proportions of subjects with depressive symptoms, poor subjective health, and unemployment status by each sociodemographic feature among the pooled observations. As the table shows, those who held negative attitudes had higher proportions of subjects with depressive symptoms (16.2% vs. 12.1%, P < 0.001), poor subjective health (15.2% vs. 10.8%, P < 0.001), and unemployment status (46.8% vs. 36.3%, P < 0.001). Also, those whose husbands had negative attitudes towards their wives’ economic participation had higher proportions of subjects with depressive symptoms (18.2% vs. 13.2%, P < 0.001), poor subjective health (20.5% vs. 11.4%, P < 0.001), and unemployment status (83.7% vs. 33.3%, P < 0.001).

**Table 2:** Proportions of depressive symptom, poor subjective health, and unemployment status among total observations.

	Depressive symptom			Poor subjective health			Unemployment status			
	Yes	No	P value	Yes	No	P value	Yes		No	P value
One's own negative attitude toward women's economic participation										
No	1117 (12.1%)	8108 (87.9%)	<0.001	993 (10.8%)	8232 (89.2%)	<0.001	3352 (36.3%)		5873 (63.7%)	<0.001
Yes	1362 (16.2%)	7069 (83.8%)		1283 (15.2%)	7148 (84.8%)		3942 (46.8%)		4489 (53.2%)	
Husband's negative attitude toward wife's economic participation										
No	1967 (13.2%)	12881 (86.7%)	<0.001	1699 (11.4%)	13149 (88.6%)	<0.001	4943 (33.3%)		9905 (66.7%)	<0.001
Yes	512 (18.2%)	2296 (81.8%)		577 (20.5%)	2231 (79.5%)		2351 (83.7%)		457 (16.3%)	
Age group										
< 40	142 (8.0%)	1624 (92.0%)	<0.001	34 (1.9%)	1732 (98.1%)	<0.001	890 (50.4%)		876 (49.6%)	<0.001
40-49	537 (9.4%)	5155 (90.6%)		236 (4.2%)	5456 (95.8%)		2197 (38.6%)		3495 (61.4%)	
50-59	713 (14.6%)	4183 (85.4%)		511 (10.4%)	4385 (89.6%)		1718 (35.1%)		3178 (64.9%)	
≥ 60	1087 (20.5%)	4215 (79.5%)		1495 (28.2%)	3807 (71.8%)		2489 (46.9%)		2813 (53.1%)	
Income (in quartile)										
Q1	1016 (22.6%)	3489 (77.4%)	<0.001	1310 (29.1%)	3195 (70.9%)	<0.001	2224 (49.4%)		2281 (50.6%)	<0.001
Q2	645 (14.2%)	3892 (85.8%)		512 (11.3%)	4025 (88.7%)		2011 (44.3%)		2526 (55.7%)	
Q3	453 (10.7%)	3768 (89.3%)		258 (6.1%)	3963 (93.9%)		1589 (37.7%)		2632 (62.3%)	
Q4	365 (8.3%)	4028 (91.7%)		196 (4.5%)	4197 (95.5%)		1470 (33.5%)		2923 (66.5%)	
Employment status										
Employed worker	549 (10.6%)	4630 (89.4%)	<0.001	268 (5.2%)	4911 (94.8%)	<0.001	0		5179 (100%)	
Self-employed	309 (13.3%)	2011 (86.7%)		215 (9.3%)	2105 (90.7%)		0		2320 (100%)	
Unpaid family worker	504 (17.6%)	2359 (82.4%)		607 (21.2%)	2256 (78.8%)		0		2863 (100%)	
Unemployed	1117 (15.3%)	6177 (84.7%)		1186 (16.3%)	6108 (83.7%)		7294 (100%)		0	
Education										
College	314 (9.8%)	2903 (90.2%)	<0.001	134 (4.2%)	3083 (95.8%)	<0.001	1451 (45.1%)		1766 (54.9%)	<0.001
High school or under	2033 (14.5%)	12,024 (85.0%)		1947 (13.8%)	12,110 (86.2%)		5693 (40.5%)		8364 (59.5%)	
None	132 (34.5%)	250 (65.5%)		195 (51.1%)	187 (48.9%)		150 (39.3%)		232 (60.7%)	
Number of children										
No	1729 (16.7%)	8606 (83.3%)	<0.001	1915 (18.5%)	8420 (81.5%)	<0.001	4214 (40.8%)		6121 (59.2%)	<0.001
One	303 (11.4%)	2357 (88.6%)		167 (6.3%)	2493 (93.7%)		1020 (38.3%)		1640 (61.7%)	
Two or more	447 (9.6%)	4214 (90.4%)		194 (4.2%)	4467 (95.8%)		2060 (44.2%)		2601 (55.8%)	
Marital satisfaction (range: 1–10)										
Mean (SD)	6.1 (1.7)	6.9 (1.5)	<0.001	6.2 (1.7)	6.9 (1.5)	<0.001	6.7 (1.6)		6.8 (1.5)	0.011
Regular physical activity										
No	1924 (14.7%)	11,112 (85.2%)	<0.001	1839 (14.1%)	11,197 (85.9%)	<0.001	5041 (38.7%)		7995 (61.3%)	<0.001
Yes	555 (12.0%)	4065 (88.0%)		437 (9.5%)	4183 (90.5%)		2253 (48.8%)		2367 (51.2%)	
Smoking										
Never	2417 (13.8%)	15,072 (86.2%)	<0.001	2251 (12.9%)	15,238 (87.1%)	0.490	7217 (41.3%)	10,272 (58.7%)		0.236
Current/past	62 (37.1%)	105 (62.9%)		25 (15.0%)	142 (85.0%)		77 (46.1%)	90 (53.9%)		
Problematic alcohol use										
No	2427 (13.9%)	15,010 (86.1%)	<0.001	2250 (12.9%)	15,187 (87.1%)	0.725	7224 (41.4%)	10,213 (58.6%)		0.006
Yes	52 (23.7%)	167 (76.3%)		26 (11.9%)	193 (88.1%)		70 (32.0%)	149 (68.0%)		

*Chi-square test or Student's t-test.

Fig. 1 shows the overall time trend of the proportion of participants who held negative attitudes towards women's economic participation and those whose husbands had negative attitudes towards their wives' economic participation. In both cases, the percentages showed a steady declining trend over the past 6 years.

**Fig. 1:**
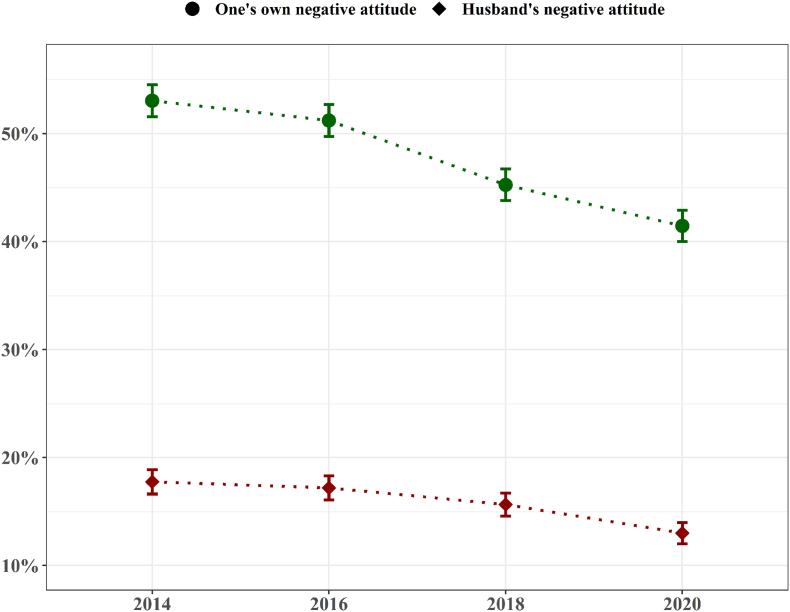
Time trend of the percentages of one's own and one's husband's negative attitude towards women/wife's economic participation by survey years.

Table 3 shows the associations between attitudes towards women's/wives' economic participation and depressive symptoms, poor subjective health, and unemployment among married women. One's own negative attitudes towards women working was associated with depressive symptoms (OR [95% CI]: 1.19 [1.09–1.31]), poor subjective health (OR [95% CI]: 1.14 [1.04–1.25]), and unemployment (OR [95% CI]: 1.10 [1.05–1.15]). Also, there were significant associations between husbands' negative attitudes towards wives' working and depressive symptoms (OR [95% CI]: 1.41 [1.23–1.60]), poor subjective health (OR [95% CI]: 1.69 [1.48–1.92]), and unemployment (OR [95% CI]: 1.80 [1.69–1.92]) among married women.

**Table 3:** Association of attitude toward women/wife's economic participation and depressive symptom, poor subjective health, and unemployment among married women [OR: Odds Ratio; CI: Confidence Interval].

Outcomes	One's own negative attitude				Husband's negative attitude			
	Crude model		Fully-adjusted model		Crude model		Fully-adjusted model	
	OR	95% CI	OR	95% CI	OR	95% CI	OR	95% CI
Depressive symptom	1.19	1.09–1.30	1.19	1.09–1.31	1.31	1.16–1.47	1.41	1.23–1.60
Poor subjective health	1.15	1.06–1.25	1.14	1.04–1.25	1.63	1.46–1.83	1.69	1.48–1.92
Unemployment	1.09	1.05–1.14	1.10	1.05–1.15	1.78	1.67–1.90	1.80	1.69–1.92

*Fully-adjust models adjusted for age, household income, education, number of children, marital satisfaction, physical activity, smoking status, problematic alcohol use, employment status (for depressive symptom and poor subjective health) and survey year.

Table 4 shows the combined effect of one's own and husband's negative attitude towards women's/wives' economic participation and depressive symptoms, poor subjective health, and unemployment status in married women. The combined group of one's own and husband's negative attitudes showed increased risks for depressive symptoms (OR [95% CI]: 1.66 [1.41–1.95])), poor subjective health (OR [95% CI]: 1.90 [1.62–2.24]), and unemployment status (OR [95% CI]: 1.94 [1.80–2.09]) compared with the reference group.

**Table 4:** Combined effect of one's own and husband negative gender role attitude toward women/wife's economic participation and depressive symptom, poor subjective health, and unemployment among married women [OR: Odds Ratio; CI: Confidence Interval].

		Depressive symptom		Poor subjective health		Unemployment	
One's own negative attitude	Husband's negative attitude	OR	95% CI	OR	95% CI	OR	95% CI
**-**	**-**	1.00	1.00–1.00	1.00	1.00–1.00	1.00	1.00–1.00
**+**	**-**	1.20	1.09–1.33	1.15	1.03–1.28	1.12	1.06–1.17
**-**	**+**	1.44	1.19–1.75	1.73	1.43–2.09	1.89	1.74–2.06
**+**	**+**	1.66	1.41–1.95	1.90	1.62–2.24	1.94	1.80–2.09

*Models adjusted for age, household income, education, number of children, marital satisfaction, physical activity, smoking status, problematic alcohol use, employment status (for depressive symptom and poor subjective health) and survey year.

Table 5 shows the cumulative effects of negative attitudes towards women's/wives' economic participation on health outcomes and employment status. For both main independent variables, a longer duration of negative attitudes was associated with a higher risk of depressive symptoms, poor subjective health, and unemployment status. Overall, the longer the duration, the stronger the effect. For instance, ≥ 3 years of one's own negative attitudes towards women's economic participation was associated with depressive symptoms (OR [95% CI]: 1.70 [1.42–2.04])), poor subjective health (OR [95% CI]: 1.28 [1.04–1.57]), and unemployment status (OR [95% CI]: 1.39 [1.22–1.58]) in married women. Similarly, ≥ 3 years of the husband's negative attitude towards the wife's economic participation was associated with depressive symptoms (OR [95% CI]: 1.32 [1.02–1.72])), poor subjective health (OR [95% CI]: 1.81 [1.40–2.35]), and unemployment status (OR [95% CI]: 9.02 [7.97–10.21]) in married women.

**Table 5:** Cumulative effect of one's own and husband's negative attitude toward women/wife's economic participation and depressive symptom, poor subjective health, and unemployment status of married women [OR: Odds Ratio; CI: Confidence Interval].

	Depressive symptom		Poor subjective health		Unemployment status	
	OR	95% CI	OR	95% CI	OR	95% CI
One's own negative attitude						
0 year	1.00	1.00–1.00	1.00	1.00–1.00	1.00	1.00–1.00
1 year	1.23	1.08–1.40	1.01	0.88–1.16	1.19	1.11–1.29
2 years	1.38	1.18–1.61	1.15	0.96–1.37	1.26	1.14–1.40
≥ 3 years	1.70	1.42–2.04	1.28	1.04–1.57	1.39	1.22–1.58
Husband's negative attitude						
0 year	1.00	1.00–1.00	1.00	1.00–1.00	1.00	1.00–1.00
1 year	1.23	1.08–1.40	1.39	1.21–1.60	2.77	2.54–3.02
2 years	1.32	1.08–1.60	1.48	1.21–1.82	4.63	4.15–5.16
≥ 3 years	1.32	1.02–1.72	1.81	1.40–2.35	9.02	7.97–10.21

*Models adjusted for age, household income, education, number of children, marital satisfaction, physical activity, smoking status, problematic alcohol use, employment status (for depressive symptom and poor subjective health) and survey year.

Table 6 presented the effect of the sequential experience of each negative attitude on depressive symptom, poor subjective health, and unemployment status of married women. Women who did not experience negative attitudes in both the previous year (*t-1*) and the concerned year (*t*) were defined as a reference group. Holding one's own negative attitudes continuously in the previous year and the concerned year increases the odds of depressive symptoms (OR [95% CI]: 1.65 [1.35–2.03]), poor subjective health (OR [95% CI]: 1.83 [1.48–2.27]), and unemployment (OR [95% CI]: 5.45 [4.83–6.15]). Similarly, when experiencing husband's negative attitudes continuously in the previous year and the concerned year, the odds of depressive symptoms (OR [95% CI]: 1.49 [1.29–1.72]), poor subjective health (OR [95% CI]: 1.26 [1.08–1.47]), and unemployment (OR [95% CI]: 1.22 [1.12–1.32]) increased.

**Table 6:** The effect of the sequential experience of each negative attitude on depressive symptom, poor subjective health, and unemployment status of married women. [OR: Odds Ratio; CI: Confidence Interval].

	Depressive symptom		Poor subjective health		Unemployment status	
	OR	95% CI	OR	95% CI	OR	95% CI
One's own negative attitude						
No → No	1.00	1.00–1.00	1.00	1.00–1.00	1.00	1.00–1.00
Yes → No	1.17	0.98–1.40	1.03	0.85–1.24	2.00	1.83–2.19
No → Yes	1.57	1.32–1.88	1.76	1.46–2.11	2.71	2.47–2.97
Yes → Yes	1.65	1.35–2.03	1.83	1.48–2.27	5.45	4.83–6.15
Husband's negative attitude						
No → No	1.00	1.00–1.00	1.00	1.00–1.00	1.00	1.00–1.00
Yes → No	1.35	1.17–1.55	1.03	0.88–1.21	1.14	1.06–1.22
No → Yes	1.45	1.24–1.70	1.20	1.03–1.41	1.18	1.09–1.28
Yes → Yes	1.49	1.29–1.72	1.26	1.08–1.47	1.22	1.12–1.32

*Variables were defined according to negative experiences in the previous year (*t-1*) and the concerned year (*t*).

**Models adjusted for age, household income, education, number of children, marital satisfaction, physical activity, smoking status, problematic alcohol use, employment status (for depressive symptom and poor subjective health) and survey year.

## 4 Discussion

Our study offers novel findings suggesting that both one's own and husband's negative attitudes towards women's/wives' economic participation are associated with depressive symptoms, poor subjective health, and unemployment status among married women. The effect was strongest when one's own and one's husband's negative attitudes were combined. Furthermore, we found cumulative effects of negative attitudes towards women's labour participation on depressive symptoms, subjective health, and unemployment in married women.

Our results are consistent with previous findings that traditional GRAs are related to poor health outcomes. Previous studies have reported that holding traditional GRAs is related to poor overall well-being (Sweeting et al., 2014), psychological distress (Read & Grundy, 2011), and suicidal behaviour (Hunt et al., 2006). In contrast, those who hold egalitarian attitudes were found to be associated with better mental health (King et al., 2019). The gender intensification hypothesis explains how traditional GRAs negatively affect health outcomes. According to the gender intensification hypothesis, those who internalise traditional gender norms (femininity or masculinity) may experience more psychological pressure, which induces gender role stress (Tolman et al., 2006). It has been demonstrated that conformity to masculine/feminine gender identity is associated with poor mental health in both sexes (Herreen et al., 2021; Rovira et al., 2022; Seidler et al., 2016). Our study found that this effect was significantly associated with depressive symptoms and poor subjective health in married women, especially when the GRAs were related to gender role differences in economic participation.

In addition, our study is the first to demonstrate that not only one's own traditional gender role identity but also the spouse's traditional GRAs towards economic participation of the wife is significantly associated with poor health outcomes in married women. In particular, negative attitudes towards wives' economic participation had an accumulating effect on depressive symptoms and the poor subjective health of married women. Emotional spousal support is a major determinant of marital satisfaction (Yedirir & Hamarta, 2015) and health (Biehle & Mickelson, 2012). In previous studies, the husband's traditional GRAs were associated with physical violence against the wife (Cheung & Choi, 2016) and poor marital quality (Amato & Booth, 1995). Overall, the results of our study might be explained by the gender role stress of married women, which could be intensified by their spouse's unsupportive attitude towards their economic participation. This gender role stress may eventually contribute to poor mental and subjective health.

Our findings confirm that women's economic participation is significantly associated with GRAs. At the national level, traditional GRAs are a major barrier to women's participation in the labour force (Fortin, 2005). Similar to a previous study (Gwak & Choi, 2015), we found that the spouse's attitudes towards economic activities have a strong association with married women's participation in economic activities. The longer the duration of the husband's negative attitudes towards his wife working, the higher the odds of his wife's unemployment status. A patriarchal and hierarchical order, influenced by Confucian culture, still has strong control over women's participation in economic activities in Korea (Park & Cho, 1995).

Finally, from a practical point of view, policy efforts are needed to alleviate the negative attitudes of both men and women towards women's participation in economic activities. One previous study found that educational programs reduced traditional GRAs towards women's economic participation (Rivera-Garrido, 2022). Also, on the national level, a cultural climate that overemphasises masculinity was associated with less egalitarian GRAs (Boehnke, 2011). In Korea, a range of policies designed to facilitate egalitarian GRAs have been implemented to support women's employment and work-family balance in recent decades (Kim, 2012). In the current study, negative perceptions of women's economic participation showed a decreasing trend in both men and women. A declining trend of traditional GRAs has also been observed in Western countries in recent decades (Knight & Brinton, 2017).

The results of this study should be interpreted with caution because of the following limitations: First, because the husband's attitude was measured indirectly by his wife, it may not have matched the actual husband's attitude. Nevertheless, how a wife perceives her husband's attitudes may be more closely associated with her health outcomes than the husband's actual attitudes. Second, the health conditions of the participants were assessed only with subjective measures. Third, since our study design did not exclude subjects with poor subjective health, depressive symptoms or unemployed status at the baseline, further studies are needed to confirm the prospective effects of GRAs. Fourth, our variables for cumulative effect of GRA are designed to sum experiences of negative attitudes until each concerned year. Therefore, the variable regarding cumulative effect may not coincide with the GRA in the concerned year, and there are cases where the negative GRAs were not consecutive. Finally, working correlation in GEE analysis is chosen according to the assumption on correlation between outcomes within participants. Therefore, the GEE result may be dependent on the correlation structure. Nevertheless, similar results were derived from sensitivity analyses using different working correlation structures.

Our study also has some notable strengths. First, to the best of our knowledge, it is the first to explore the possible health effects of spouses’ gender role attitudes. Second, our results were drawn from samples that represent Korean women because the subjects were sampled systemically. Thus, our findings have strength in terms of generalisability. Third, our study investigated how the effects vary when GRAs of wives and husband coincide or conflict. We found that ORs of poor subjective health, depressive symptom and unemployment were also affected by the change and accumulative experience of negative GRAs.

## 5 Conclusion

Our results show that one's own and one's husband's attitudes towards women's economic participation affects not only the employment status of married women but also their mental and subjective health. Policymakers should implement measures that promote positive attitudes towards women's economic activities.

## Ethics statement

This study was reviewed and approved by the Institutional Review Board of Yonsei Health System (approval number: 4-2022-0691).

## Funding

No external funding was received.

## CRediT authorship contribution statement

**Seong-Uk Baek:** Conceptualization, Formal analysis, Data curation, Investigation, Writing – original draft. **Jin-Ha Yoon:** Investigation, Supervision. **Jong-Uk Won:** Conceptualization, Writing – review & editing, Supervision.

## Declaration of competing interest

None.

## Figures and Tables

**figs1:**
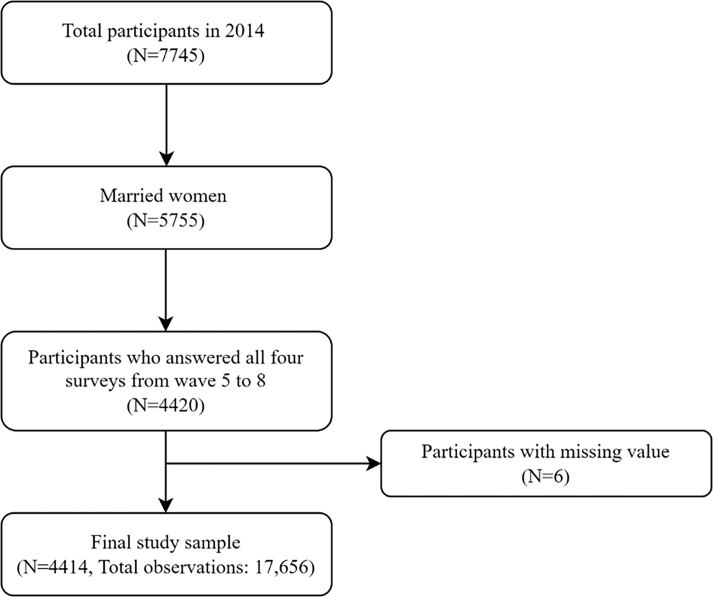


## Data Availability

I have shared the link to my data at Attach File step
