# Supplementary material for: Associations between wives' and husbands' attitudes towards women's economic participation and depressive symptoms, poor subjective health, and unemployment status in married women: A Korean longitudinal study (2014–2020)
Source: SSM Popul Health. 2022 Oct 22;20:101275. doi: 10.1016/j.ssmph.2022.101275 (PMC9637806; doi:10.1016/j.ssmph.2022.101275)
Supplement: Multimedia component 1 [file mmc1.docx]

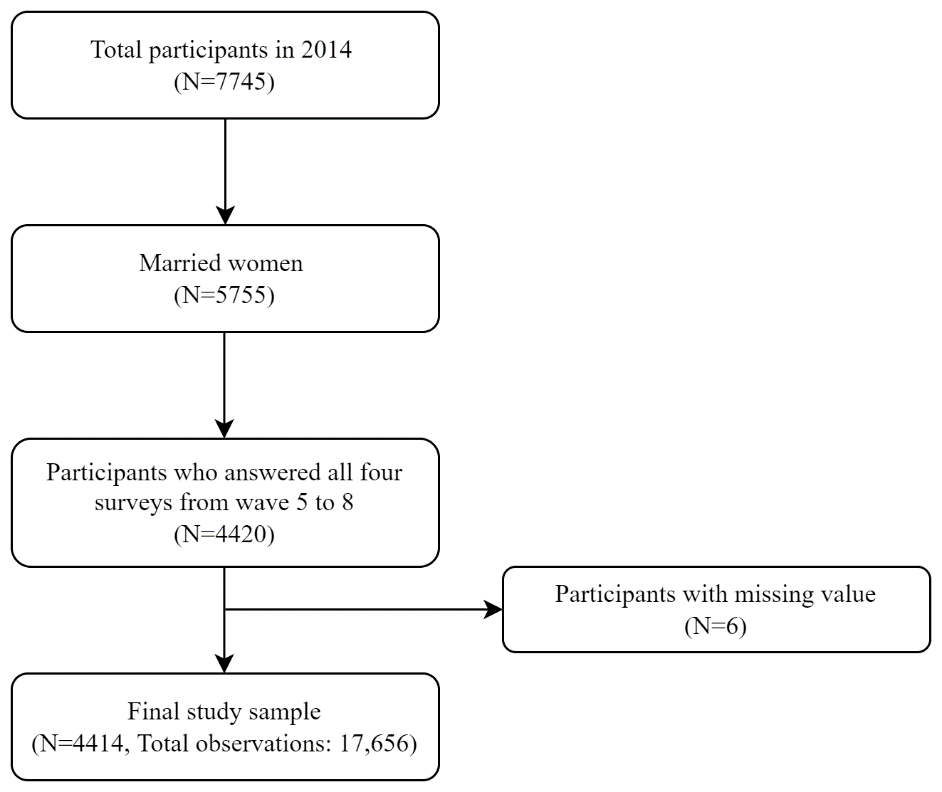


**Figure S1** Flowchart of study sample selection

**Table S1.** Sociodemographic features among study samples by each survey year

|  | | **Year** | | | | | | | |  | |
| --- | --- | --- | --- | --- | --- | --- | --- | --- | --- | --- | --- |
|  | | **2014 (WAVE 5)**  **N=4414** | | **2016 (WAVE 6)**  **N=4414** | | **2018 (WAVE 7)**  **N=4414** | | **2020 (WAVE 8)**  **N=4414** | | **Overall**  **N=17,656** | |
| Depressive symptom | |  | |  | |  | |  | |  | |
| No | | 3813 (86.4%) | | 3873 (87.7%) | | 3795 (86.0%) | | 3696 (83.7%) | | 15,177 (86.0%) | |
| Yes | | 601  (13.6%) | | 541  (12.3%) | | 619  (14.0%) | | 718  (16.3%) | | 2479  (14.0%) | |
| Poor subjective health | |  | |  | |  | |  | |  | |
| No | | 3930 (89.0%) | | 3848 (87.2%) | | 3864 (87.5%) | | 3738 (84.7%) | | 15,380 (87.1%) | |
| Yes | | 484  (11.0%) | | 566  (12.8%) | | 550  (12.5%) | | 676  (15.3%) | | 2276  (12.9%) | |
| Employment status | |  | |  | |  | |  | |  | |
| Employed worker | | 1212 (27.5%) | | 1261 (28.6%) | | 1331 (30.2%) | | 1375 (31.2%) | | 5179  (29.3%) | |
| Self-employed | | 588  (13.3%) | | 598  (13.5%) | | 585  (13.3%) | | 549  (12.4%) | | 2320  (13.1%) | |
| Unpaid family worker | | 740  (16.8%) | | 740  (16.8%) | | 708  (16.0%) | | 675  (15.3%) | | 2863  (16.2%) | |
| Unemployed | | 1874 (42.5%) | | 1815 (41.1%) | | 1790 (40.6%) | | 1815 (41.1%) | | 7294  (41.3%) | |
| Age group | |  | |  | |  | |  | |  | |
| < 40 | | 776  (17.6%) | | 502  (11.4%) | | 317  (7.2%) | | 171  (3.9%) | | 1766  (10.0%) | |
| 40-49 | | 1499 (34.0%) | | 1533 (34.7%) | | 1410 (31.9%) | | 1250 (28.3%) | | 5692  (32.2%) | |
| 50-59 | | 1147 (26.0%) | | 1163 (26.3%) | | 1263 (28.6%) | | 1323 (30.0%) | | 4896  (27.7%) | |
| ≥ 60 | | 992  (22.5%) | | 1216 (27.5%) | | 1424 (32.3%) | | 1670 (37.8%) | | 5302  (30.0%) | |
| Income (in quartile) | |  | |  | |  | |  | |  | |
| Q1 | | 1183 (26.8%) | | 1091 (24.7%) | | 1078 (24.4%) | | 1153 (26.1%) | | 4505  (25.5%) | |
| Q2 | | 1360 (30.8%) | | 1180 (26.7%) | | 1069 (24.2%) | | 928  (21.0%) | | 4537  (25.7%) | |
| Q3 | | 1063 (24.1%) | | 1117 (25.3%) | | 1054 (23.9%) | | 987  (22.4%) | | 4221  (23.9%) | |
| Q4 | | 808  (18.3%) | | 1026 (23.2%) | | 1213 (27.5%) | | 1346 (30.5%) | | 4393  (24.9%) | |
| Education | |  | |  | |  | |  | |  | |
| College | | 799  (18.1%) | | 803  (18.2%) | | 808  (18.3%) | | 807  (18.3%) | | 3217  (18.2%) | |
| High school or under | | 3519 (79.7%) | | 3515 (79.6%) | | 3511 (79.5%) | | 3512 (79.6%) | | 14,057 (79.6%) | |
| None | | 96  (2.2%) | | 96  (2.2%) | | 95  (2.2%) | | 965  (2.2%) | | 382  (2.2%) | |
| Number of children | |  | |  | |  | |  | |  | |
| No | | 2232 (50.6%) | | 2436 (55.2%) | | 2678 (60.7%) | | 2989 (67.7%) | | 10,335 (58.5%) | |
| One | | 708  (16.0%) | | 675  (15.3%) | | 672  (15.2%) | | 605  (13.7%) | | 2660  (15.1%) | |
| Two or more | | 1474 (33.4%) | | 1303 (29.5%) | | 1064 (24.1%) | | 820  (18.6%) | | 4661  (26.4%) | |
| Relationship with husband | |  | |  | |  | |  | |  | |
| Mean (SD) | | 6.9 (1.7) | | 6.8 (1.6) | | 6.7 (1.5) | | 6.7 (1.4) | | 6.8 (1.5) | |
| Regular physical activity | |  | |  | |  | |  | |  | |
| No | | 3270 (74.1%) | | 3313 (75.1%) | | 3152 (71.4%) | | 3301 (74.8%) | | 13,036 (73.8%) | |
| Yes | | 1144 (25.9%) | | 1101 (24.9%) | | 1262 (28.6%) | | 1113 (25.2%) | | 4620  (26.2%) | |
| Smoking | |  | |  | |  | |  | |  | |
| Never | | 4358 (98.7%) | | 4379 (99.2%) | | 4363 (98.8%) | | 4389 (99.4%) | | 17,489 (99.1%) | |
| Current/past | | 56  (1.3%) | | 35  (0.8%) | | 51  (1.2%) | | 25  (0.6%) | | 167  (0.9%) | |
| Problematic alcohol use | |  | |  | |  | |  | |  | |
| No | | 4349 (98.5%) | | 4345 (98.4%) | | 4372 (99.0%) | | 4371 (99.0%) | | 17,437 (98.8%) | |
| Yes | | 65  (1.5%) | | 69  (1.6%) | | 42  (1.0%) | | 43  (1.0%) | | 219  (1.2%) | |

**Table S2.** Baseline characteristics of included and excluded participants

|  | Included  (N=4414) | Excluded  (N=1341) | P value |
| --- | --- | --- | --- |
| Age group |  |  |  |
| < 40 | 776 (17.6%) | 282 (21.0%) | 0.014 |
| 40-49 | 1499 (34.0%) | 446 (33.3%) |  |
| 50-59 | 1147 (26.0%) | 308 (23.0%) |  |
| ≥ 60 | 992 (22.5%) | 305 (22.7%) |  |
| Income (in quartile) |  |  |  |
| Q1 | 1183 (26.8%) | 379 (28.3%) | 0.442 |
| Q2 | 1282 (29.0%) | 383 (28.6%) |  |
| Q3 | 1128 (25.6%) | 318 (23.7%) |  |
| Q4 | 821 (18.6%) | 261 (19.5%) |  |
| Unemployment |  |  |  |
| Employed worker | 1212 (27.5%) | 366 (27.3%) | < 0.001 |
| Self-employed | 588 (13.3%) | 143 (10.7%) |  |
| Unpaid family worker | 740 (16.8%) | 141 (10.5%) |  |
| Unemployed | 1874 (42.5%) | 691 (51.5%) |  |
| Education |  |  |  |
| College | 799 (18.1%) | 278 (20.8%) | < 0.001 |
| High school or under | 3519 (79.7%) | 1011 (75.4%) |  |
| None | 96 (2.2%) | 50 (3.7%) |  |
| Missing | 0 (0.0%) | 2 (0.1%) |  |
| Number of children |  |  |  |
| No | 2232 (50.6%) | 635 (47.4%) | 0.074 |
| One | 708 (16.0%) | 243 (18.1%) |  |
| Two or more | 1474 (33.4%) | 463 (34.5%) |  |
| Relationship with husband (range: 1-10) |  |  |  |
| Mean (SD) | 6.9 (1.7) | 6.6 (1.9) | < 0.001 |
| Regular physical activity |  |  |  |
| No | 3270 (74.1%) | 992 (74.0%) | 0.965 |
| Yes | 1144 (25.9%) | 349 (26.0%) |  |
| Smoking |  |  |  |
| Never | 4358 (98.7%) | 1327 (99.0%) | 0.606 |
| Current/past | 56 (1.3%) | 14 (1.0%) |  |
| Problematic alcohol use |  |  |  |
| No | 4349 (98.5%) | 1319 (98.4%) | 0.754 |
| Yes | 65 (1.5%) | 22 (1.6%) |  |

**Table S3.** Association of attitude toward women/wife’s economic participation and depressive symptom, poor subjective health and unemployment among married women using fully-adjusted model based on exchangeable and unstructed working correlation [OR: Odds Ratio; CI: Confidence Interval, WC: Working Correlation]

|  | One’s own negative attitude | | | |  | Husband’s negative attitude | | | |
| --- | --- | --- | --- | --- | --- | --- | --- | --- | --- |
|  | Exchangeable WC | | Unstructured WC | |  | Exchangeable WC | | Unstructured WC | |
| Outcomes | OR | 95% CI | OR | 95% CI |  | OR | 95% CI | OR | 95% CI |
| Depressive symptom | 1.20 | 1.10-1.32 | 1.19 | 1.09-1.30 |  | 1.44 | 1.27-1.65 | 1.42 | 1.25-1.62 |
| Poor subjective health | 1.16 | 1.06-1.28 | 1.15 | 1.05-1.27 |  | 1.70 | 1.50-1.93 | 1.70 | 1.50-1.93 |
| Unemployment | 1.14 | 1.09-1.19 | 1.12 | 1.07-1.17 |  | 2.32 | 2.16-2.48 | 2.03 | 1.91-2.17 |

*****Fully-adjust models adjusted for age, household income, education, number of children, marital satisfaction, physical activity, smoking status, problematic alcohol use, employment status (for depressive symptom and poor subjective health) and survey year

**Table S4.** Combined effect of one’s own and husband negative gender role attitude toward women/wife’s economic participation and depressive symptom, poor subjective health, and unemployment among married women based on exchangeable and unstructed working correlation [OR: Odds Ratio; CI: Confidence Interval, WC: Working Correlation]

|  | | Depressive symptom | |  | Poor subjective health | |  | Unemployment | |
| --- | --- | --- | --- | --- | --- | --- | --- | --- | --- |
| One’s own negative attitude | Husband’s negative attitude | Exchangeable WC | Unstructured  WC |  | Exchangeable WC | Unstructured  WC |  | Exchangeable WC | Unstructured  WC |
|  |  | OR [95% CI] | OR [95% CI] |  | OR [95% CI] | OR [95% CI] |  | OR [95% CI] | OR [95% CI] |
| **-** | **-** | 1.00 [1.00-1.00] | 1.00 [1.00-1.00] |  | 1.00 [1.00-1.00] | 1.00 [1.00-1.00] |  | 1.00 [1.00-1.00] | 1.00 [1.00-1.00] |
| **+** | **-** | 1.22 [1.10-1.35] | 1.20 [1.09-1.33] |  | 1.18 [1.06-1.32] | 1.17 [1.05-1.30] |  | 1.16 [1.10-1.22] | 1.14 [1.08-1.19] |
| **-** | **+** | 1.49 [1.24-1.80] | 1.46 [1.21-1.76] |  | 1.77 [1.48-2.13] | 1.77 [1.47-2.12] |  | 2.46 [2.24-2.69] | 2.14 [1.97-2.33] |
| **+** | **+** | 1.72 [1.46-2.02] | 1.68 [1.43-1.97] |  | 1.95 [1.66-2.30] | 1.94 [1.65-2.28] |  | 2.56 [2.36-2.78] | 2.22 [2.06-2.39] |

*****Fully-adjust models adjusted for age, household income, education, number of children, marital satisfaction, physical activity, smoking status, problematic alcohol use, employment status (for depressive symptom and poor subjective health) and survey year

**Table S5.** Combined effect of one’s own and husband’s negative attitude toward women/wife’s economic participation and depressive symptom, poor subjective health and unemployment status of marrieds women based on exchangeable and unstructed working correlation [OR: Odds Ratio; CI: Confidence Interval, WC: Working Correlation]

|  | Depressive symptom | |  | Poor subjective health | |  | Unemployment status | |
| --- | --- | --- | --- | --- | --- | --- | --- | --- |
|  | Exchangeable WC | Unstructured  WC |  | Exchangeable WC | Unstructured  WC |  | Exchangeable WC | Unstructured  WC |
|  | OR [95% CI] | OR [95% CI] |  | OR [95% CI] | OR [95% CI] |  | OR [95% CI] | OR [95% CI] |
| One’s own negative attitude |  | |  |  | |  |  | |
| 0 year | 1.00 [1.00-1.00] | 1.00 [1.00-1.00] |  | 1.00 [1.00-1.00] | 1.00 [1.00-1.00] |  | 1.00 [1.00-1.00] | 1.00 [1.00-1.00] |
| 1 year | 1.24 [1.09-1.41] | 1.23 [1.08-1.40] |  | 1.03 [0.90-1.19] | 1.03 [0.90-1.19] |  | 1.20 [1.11-1.29] | 1.20 [1.12-1.30] |
| 2 years | 1.37 [1.18-1.60] | 1.37 [1.18-1.60] |  | 1.15 [0.97-1.37] | 1.15 [0.97-1.37] |  | 1.24 [1.12-1.37] | 1.26 [1.14-1.39] |
| ≥ 3 years | 1.72 [1.43-2.05] | 1.70 [1.42-2.04] |  | 1.25 [1.02-1.53] | 1.24 [1.01-1.51] |  | 1.32 [1.16-1.50] | 1.38 [1.22-1.56] |
| Husband’s negative attitude |  | |  |  |  |  |  | |
| 0 year | 1.00 [1.00-1.00] | 1.00 [1.00-1.00] |  | 1.00 [1.00-1.00] | 1.00 [1.00-1.00] |  | 1.00 [1.00-1.00] | 1.00 [1.00-1.00] |
| 1 year | 1.22 [1.07-1.39] | 1.23 [1.08-1.39] |  | 1.41 [1.23-1.62] | 1.41 [1.23-1.61] |  | 2.79 [2.55-3.05] | 2.85 [2.62-3.10] |
| 2 years | 1.29 [1.06-1.57] | 1.30 [1.07-1.58] |  | 1.44 [1.18-1.77] | 1.45 [1.18-1.78] |  | 3.79 [3.36-4.26] | 4.46 [3.99-4.97] |
| ≥ 3 years | 1.25 [0.97-1.62] | 1.28 [0.99-1.66] |  | 1.73 [1.35-2.22] | 1.75 [1.36-2.24] |  | 5.48 [4.84-6.21] | 7.78 [6.89-8.78] |

*****Models adjusted for age, household income, education, number of children, marital satisfaction, physical activity, smoking status, problematic alcohol use, employment status (for depressive symptom and poor subjective health) and survey year

**Table S6.** The effect of the sequential experience of each negative attitude on depressive symptom, poor subjective health, and unemployment status of married women based on exchangeable and unstructed working correlation [OR: Odds Ratio; CI: Confidence Interval, WC: Working Correlation]

|  | Depressive symptom | |  | Poor subjective health | |  | Unemployment status | |
| --- | --- | --- | --- | --- | --- | --- | --- | --- |
|  | Exchangeable WC | Unstructured  WC |  | Exchangeable WC | Unstructured  WC |  | Exchangeable WC | Unstructured  WC |
|  | OR [95% CI] | OR [95% CI] |  | OR [95% CI] | OR [95% CI] |  | OR [95% CI] | OR [95% CI] |
| One’s own negative attitude |  | |  |  | |  |  | |
| No → No | 1.00 [1.00-1.00] | 1.00 [1.00-1.00] |  | 1.00 [1.00-1.00] | 1.00 [1.00-1.00] |  | 1.00 [1.00-1.00] | 1.00 [1.00-1.00] |
| Yes → No | 1.13 [0.94-1.35] | 1.15 [0.96-1.37] |  | 1.03 [0.85-1.24] | 1.03 [0.86-1.24] |  | 1.93 [1.76-2.11] | 1.99 [1.82-2.17] |
| No → Yes | 1.60 [1.34-1.91] | 1.59 [1.33-1.90] |  | 1.80 [1.50-2.16] | 1.79 [1.50-2.14] |  | 2.84 [2.57-3.13] | 2.75 [2.51-3.02] |
| Yes → Yes | 1.66 [1.36-2.03] | 1.66 [1.35-2.03] |  | 1.77 [1.43-2.19] | 1.78 [1.44-2.20] |  | 5.30 [4.71-5.97] | 5.51 [4.89-6.20] |
| Husband’s negative attitude |  | |  |  |  |  |  | |
| No → No | 1.00 [1.00-1.00] | 1.00 [1.00-1.00] |  | 1.00 [1.00-1.00] | 1.00 [1.00-1.00] |  | 1.00 [1.00-1.00] | 1.00 [1.00-1.00] |
| Yes → No | 1.35 [1.17-1.55] | 1.34 [1.17-1.55] |  | 1.00 [0.86-1.17] | 1.01 [0.86-1.18] |  | 1.14 [1.06-1.22] | 1.14 [1.07-1.23] |
| No → Yes | 1.47 [1.26-1.71] | 1.46 [1.25-1.70] |  | 1.20 [1.02-1.40] | 1.19 [1.02-1.40] |  | 1.21 [1.12-1.30] | 1.20 [1.11-1.29] |
| Yes → Yes | 1.49 [1.29-1.72] | 1.48 [1.28-1.71] |  | 1.24 [1.07-1.45] | 1.24 [1.06-1.44] |  | 1.23 [1.13-1.34] | 1.23 [1.13-1.34] |

*****Variables were defined according to negative experiences in the previous year and the concerned year.

******Models adjusted for age, household income, education, number of children, marital satisfaction, physical activity, smoking status, problematic alcohol use, employment status (for depressive symptom and poor subjective health) and survey year
